# Magnitude of intestinal parasitic infections and its determinants among HIV/AIDS patients attending at antiretroviral treatment centers in East and West Gojam Zones, Northwest, Ethiopia: institution based cross-sectional study

**DOI:** 10.1186/s12981-024-00618-3

**Published:** 2024-05-16

**Authors:** Mengistu Endalamaw, Abel Alemneh, Gashaw Azanaw Amare, Abebe Fenta, Habtamu Belew

**Affiliations:** Department of Medical Laboratory Science, College of Health Sciences, Debre Markos University, P.O. Box 269, Debre Markos, Ethiopia

**Keywords:** Intestinal parasite, Predictors, HIV/ADIS, Ethiopia

## Abstract

**Background:**

Intestinal parasitic infections (IP) are a major source of morbidity in people living with Human immunodeficiency virus (HIV), particularly in resource-limited settings, mostly as a result of high viral load. Hence, this study aimed to investigate the magnitude of intestinal parasitic infections and its determinants among patients with HIV/AIDS attending public health facilities in East and West Gojam Zones in Ethiopia.

**Methods:**

Institution-based cross-sectional study was conducted on 327 people living with HIV visiting public health facilities from December 2022 to May 2023. A simple random sampling technique was used to recruit participants. Face-to-face interviews were used to collect socio-demographics and determinants. The fresh stool was collected from each patient, transported, and tested in accordance with laboratory standard operating procedures of wet mount, formol-ether concentration technique, and modified acid-fast staining. Data were entered and analyzed in the statistical package for Social Science (SPSS) version 20. A 95% CI with p-value < 0.05 was considered statistically significant.

**Results:**

The overall prevalence of IP in patients with HIV/AIDS was 19.3% (63/327). *Hookworm* was the most identified parasite 33.3% (21/63) followed by *E.histolytica* 17% (11/63) and G.*lamblia* 14.3% (9/63). Parasitic infections were significantly higher among viral load > 1000cps/ml (*p* = 0.035), WHO stage 4 (*p* = 0.002), CD4 < 200 cell/mm^3^ (*p* = 0.001), and bare foot walking (*p* = 0.001).

**Conclusion:**

IP infections are moderately high among patients with HIV/AIDS in the study area. The proportion of parasites was greatly affected by high viral load, WHO stage 4, CD4 < 200 cell/mm^3^, and being barefoot; this gives valuable insight to health professionals, health planners and community health workers. As a result, viral load monitoring, and WHO stage controlling were periodically assessed in patients with HIV/AIDS. Health education, awareness creation, routine stool examination, and environmental hygiene were regularly advocated to increase the life of patients with HIV/AIDS.

## Introduction

Human immune deficiency virus HIV) is the most fatal virus causing the disease called acquired immunodeficiency syndrome (AIDS), this disease is known to depress the immune system of the infected individual, which favors other infectious organisms to cause disease including IP infections (1). Parasitic infections are the frequent cause of morbidity and mortality associated with AIDS patients by causing diarrhea, *Cryptosporidium parvum* and *Isospora belli* are the most common opportunistic infectious parasites (2). In Ethiopia, HIV/AIDS remains a significant public health concern, with a high prevalence and substantial impact on the population (3). In Ethiopia, the total number of people with HIV/AIDS is 617,921, comprising 235,550 males and 382,371 females across all age groups, with 40, 528 children (4). Despite progress in the prevention and treatment of HIV/AIDS, co-infections with intestinal parasites continue to pose a significant public burden on affected individuals (5). Ongoing replication of HIV leads to a constant state of immune activation that persists during the chronic phase. This immune activation is characterized by heightened activity of immune cells and the release of proinflammatory cytokines. It occurs due to the effects of various HIV gene products as well as the immune response triggered by continuous HIV replication. Furthermore, the depletion of mucosal CD4 + T cells in the early stage of the disease disrupts the immune surveillance system of the intestinal barrier, contributing to immune activation (6, 7). Intestinal parasite infections have been shown to exacerbate the immunosuppression associated with HIV/AIDS, resulting in increasing susceptibility of opportunistic infections leading to decreased life expectancy rates among people living with HIV/ADIS (8).

The burden of IP among HIV patients in Africa was found to be more prevalent from 20.9% to 65.3% (9–13), and in Ethiopia, the magnitude of IP in people with HIV from 2011 to 2020 was 39.15% (14). The East and West Gojam Zones, located in the Northwest region of Ethiopia, are known for the high prevalence of HIV/AIDS (15). However, limited research has been conducted to assess the magnitude of intestinal parasite infections among patients with HIV in East and West Gojam Zones. Understanding the prevalence and associated factors of intestinal parasite infections in this vulnerable population is crucial for developing targeted interventions and improving the overall health outcomes of patients with HIV (5).

This research aims to determine the magnitude of intestinal parasite infections and their associated factors among patients with HIV attending antiretroviral therapy in the East and West Gojam Zones of Northwest Ethiopia. By assessing the prevalence of intestinal parasite infections and identifying the factors contributing to their occurrence, this study seeks to provide valuable insights for policymakers, healthcare providers, and researchers working toward the control and prevention of these infections.

## Methods

### Study area and study design

A cross-sectional study was conducted from December 2022 to May 2023 at selected public health institutions from East and West Gojam zones in North-western Ethiopia. Debre Markos Comprehensive Specialized Hospital (DMCSH) provides service for 1340, Yejube Primary Hospital (YPH) 385 and Lumamie Primary Hospital (LPH) 221 from the East Gojam zone, and Finote Selam General Hospital (FSGH) 620 from the West Gojam.

### Study population

All HIV-positive patients who attended ART clinics at selected public health facilities.

### Exclusion criteria

People living with HIV/AIDS who had a mental illness, because they were unable to provide consent and patients who received anti-parasitic drugs within two weeks were excluded.

### Sample size determination

The sample size was determined by using the single population proportion formula, with the formula n = (Z_a/2_)^2^*P (1-P)/d^2^, where n is the minimum required sample size, P is the prevalence of intestinal parasite among HIV patients from a previous study (*p* = 24.2%) (16), d is the marginal error between the sample and the population (*d* = 0.05), and Z is the critical value at 95% certainty (1.96). Then *n* = (1.96)^2^(0.242) (0.799)/(0.05)^2^ = 297.

The final sample size, including a 10% non-responding rate, was 327.

### Sampling technique and sampling procedure

A simple random sampling with a computer-generated technique was used to select study participants in each selected health institution and proportionally allocated in each facility. From DMCSH (*n* = 171), FSGH (*n* = 79), LPH (*n* = 49), and YPH (*n* = 28) were collected.

### Data collection, processing, and analysis

#### Data collection

Socio-demographic and clinical data were collected through a face-to-face interviewer-administered structured questionnaire.

#### Stool sample collection

A fresh stool sample was collected with clean and wide-mouthed plastic containers and was preserved by formalin for the direct wet mount, formal-ether concentration, and modified acid-fast staining, if not processed immediately.

#### Stool sample direct microscopy

A fresh stool sample was collected in labeled cups from all study participants and a direct saline wet mount of each sample was done at the laboratory for motile trophozoite, ova, cyst, and larval stages of intestinal parasites. The wet mounts were examined under the light microscope at 10X and 40X objectives.

#### Formol-Ether concentration technique

The formol-ether sedimentation technique was performed from fresh and preserved stool samples as follows. A suspension of stool was made from a gram of stool sample with 7 ml of formalin in a 15 ml conical centrifuge tube and filtered through the sieve, then 3 ml of diethyl ether was added into and centrifuged at 3200 rpm for 3 min. After that, the smear was made from the sediment for microscopic examination under 10 × and 40 × objectives.

#### Modified Ziehl Neelson method

A small portion of the fresh stool sample was processed for the detection of opportunistic parasites using the modified Ziehl-Neelson method. A thin smear was prepared directly from the sediment of concentrated stool and allowed to air dry. Then the slide was fixed with methanol for 5 min and it was stained with 1% carbol-fuchsine for 30 min. After washing the slide in tap water, the slide was decolorized with 1% acid alcohol for 2 min and stained in 0.5% methylene blue for 1 min. The slide was then washed in tap water and observed under a light microscope with a magnification of 1000X.

#### Data analysis

Data was entered and analyzed by using the SPSS version 20 software package. Univariate and multivariate logistic regression were used to assess the associations of independent variables and dependent variables. All variables with p-values less than 0.25 in the Univariate analysis were candidates for multivariable logistic regression analysis to resolve the confounding effects. The association between independent variables and dependent variables was considered to be statistically significant only if the *P* value was less than < 0.05 at a 95% confidence level.

## Result

### Socio-demographic characteristics of the study population

A total of 327 individuals living with HIV/AIDS were enrolled in the study. Most of the participants, 52% (*n* = 170) were urban residents. The majority (54.2%) of the study participant’s age group was from 21 to 40 years and 63% were married. Regarding the educational status of the study participants, 38.2% were illiterate and 31.8% had high school (Table 1).

**Table 1:** Socio-demographic characteristics of people living with HIV AIDS at selected public health facilities, in Ethiopia from December 2022 and May 2023

Variables		Frequency	Percentage
Gender	Female	177	54.1
	Male	150	45.9
Address	Urban	171	52.3
	Rural	156	47.7
Age group	< 10	29	8.8
	11–20	51	15.6
	21–30	94	28.8
	31–40	83	25.4
	41–50	32	9.7
	51–60	21	6.5
	> = 61	17	5.2
Marital status	Married	206	63.0
	Single	48	14.7
	Widowed	42	12.8
	Divorced	31	9.5
Educational status	Unable to read and write	125	38.2
	Primary	98	30.0
	High school and above	104	31.8
Income	< 500	19	5.8
	500–1000	125	38.2
	> = 1000	183	56.0

### Prevalence and distribution of intestinal parasites

**Fig. 1:**
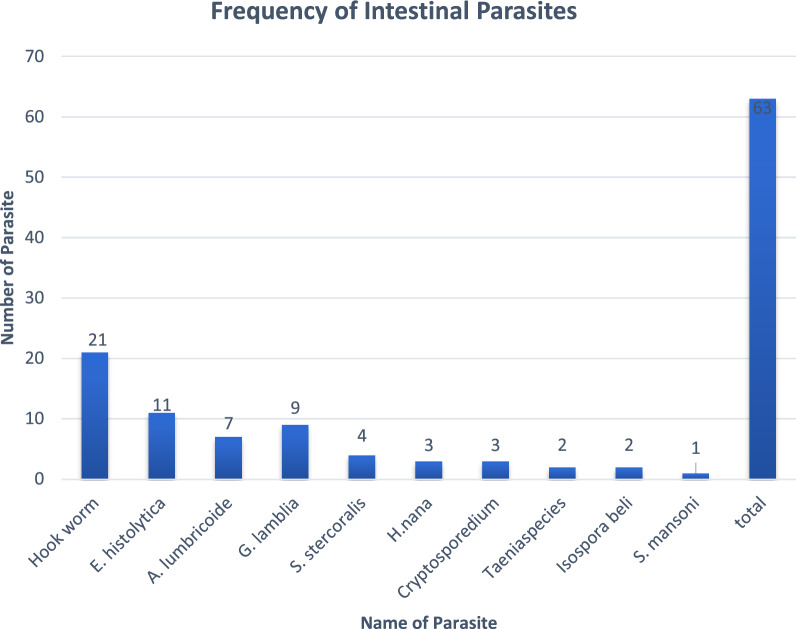
Intesting parasite distribution among people living with HIV/AIDS in West and East Gojam, Ethiopia, 2023

The overall prevalence of IP among people living with HIV/AIDS was 19.3% (*n* = 63). Of which 33.3% (*n* = 21) were *Hookworm*, which was the highest prevalent followed by 17% (*n* = 11) *E. histolytica*. Distribution of helminthic parasites 58.7% (*n* = 37) were more prevalent than protozoan parasites 41.3% (*n* = 26) (Fig. 1).

### Association of intestinal parasites with socio-demographic and other risk factors

Out of the total 63 IP-positive participants, 69.8% (*n* = 44) were female, and 30.2% (*n* = 19) were male. The majority, 28.6% of infected individuals were aged 31–40, and individuals aged 51–60 had less probability of being infected with IP (6.3%), regarding the marriage status the majority of infected participants 71.4% (*n* = 45) were married. Of patients with HIV diagnosed with IP infection, 58.7% (n = 37) had latrines, and 45.9% (n = 17) of participants used them always, whereas 35.2% (n = 13) didn’t use them at all. Of co-infected 98.4% had regular hand-washing habits before meals (Table 2).

**Table 2:** Magnitude of Intestinal parasitic infection in different groups of HIV/ADIS patients at East and West Gojam, Ethiopia, December 2022 and May 2023

Characteristic		Intestinal parasite		COR (95CL)	p-value	AOR (95CL)	P- value
		Positive N(%)	Negative N(%)				
Sex	Male	19(30.2)	131(49.6)	1			
	Female	44(69.8)	133(46.6)	2.28(2.26–4.11)	0.06	1.58(0.07–36.9)	0.86
Age	1–10	8(12.7)	21(8)	1.83 (0.51–6.5)	0.34	0.25(0.02–3.03)	0.28
	11–20	7(11.1)	44(16.7)	4.4(1.25–15.5)	0.2	0.22(0.02–2.2)	0.201
	21–30	13(20.6)	81(30.7)	4.36(1.4–13.4)	0.11	0.27(0.03–2.05)	0.209
	31–40	18(28.6)	65(24.6)	2.5(0.84–7.6)	0.98	0.35(0.04–2.67)	0.31
	41–50	6(9.5)	26(9.8)	3.03(0.81–11.2)	0.97	0.32(0.03–3.1)	0.33
	51–60	4(6.3)	17(6.4)	2.97(0.69–12.7)	0.142	1.09(0.10–10.9)	0.94
	> = 61	7(11.1)	10(3.8)	1			
Address	Urban	32(50.8)	139(52.7)	0.92(0.53–1.60)	0.79		
	Rural	31(49.2)	125(47.3)	1			
Educational status	Unable to read and write	39(61.9)	86(32.6)	0.18(0.81–0.415)	0	1.41(0.04–45.6)	0.84
	Primary	16(25.4)	82(31.1)	0.427(0.17–1.04)	0.63	8.52(0.11–61.9)	0.32
	High school and above	8(12.7)	96(36.4)	1			
Marital status	Married	45(71.4)	161(61)	1.27(0.59–2.72)	0.54	0.14(0.006–3.8)	0.25
	Unmarried	3(4.8)	45(17)	5.32(1.37–20.6)	0.016	0.09(0.002–5.3)	0.24
	Divorced	11(17.5)	31(11.7)	2.3(0.68–8.4)	0.173		
	Windowed	4(6.3)	27(10.2)	1			
Income (ETB)	< 500	9(14.3)	10(3.8)	0.32(0.12–0.84)	0.21		
	500–100	13(20.6)	112(42.4)	2.48(1.27–4.8)	0.08	0.703(0.017–28.2)	0.85
	> 1000	41(65.1)	142(53.8)	1			
Availability of latrine	Yes	37(58.7)	206(78)	1			
	No	26(41.3)	58(22)	2.49(1.39–4.45)	0.02	1.21(1.10–3.4)	0.03**
Habit of latrine usage	always	17(45.9)	102(46.5)	1.81(0.71–4.5)	0.21		
	sometimes	7(18.9)	76(36.9)	0.35(0.15–0.82)	0.16	0.37(0.13–4.53)	0.67
	Not at all	13(35.1)	28(13.6)	1			
Contact with soil	Yes	23(36.5)	81(30.7)	1.299(1.17–2.31)	0.023	0.60(0.13–2.7)	0.51
	No	40(63.5)	183(69.3)	1			
Diarrhea	Yes	44(69.8)	82(31.1)	0.19(0.107–0.354)	0	4.5(0.29–71.4)	0.28
	No	19(30.2)	182(68.9)	1			
How long on diarrhea	< 1 month	36(80)	71(87.7)	1.77(0.86–4.7)	0.254		
	> 1 month	9(20)	10(12.3)	1			
Viral load	TND	32(50.8)	237(90.1)	1			
	20-1000cps/ml	17(27)	21(8)	0.167(0.80–0.349)	0.001	2.37(1.92–20.1)	0.035**
	> 1000cps/ml	14(22.2)	5(1.9)	0.048(0.016–0.14)	0.001	0.15(0.02–1.19)	0.68
WHO stage of HIV/ADIS	Stage 1	39(61.9)	207(78.4)	1			
	Stage 2	14(22.2)	42(15.9)	0.56(0.28–1.13)	0.1	0.92(0.09–2.9)	0.46
	Stage 3	3(4.8)	1(0.4)	0.063(0.006–0.619)	0.018	0.69(0.06–7.9)	0.77
	Stage 4	7(11.1)	14(5.3)	0.37(0.14–0.99)	0.049	3.83(1.23, 11.54)	0.002**
CD4 category	< 200	25(39.7)	28(10.6)	6.69 (3.4–13.2)	0.001	5.7 (2.77–11.7)	0.001**
	200–500	12(19)	41(15.5)	3.05 (1.3–7.06)	0.009	4.6 (1.8–11.7)	0.001**
	> 500	26(41.3)	195(73.9)	1		1	
Shoe wear	Yes	23(36.5)	140(53)	7.8 (3.3–18.4)	0.001	6.6 (2.7–16.4)	0.001**
	No	40(63.5)	124(47)		1	1	

*TND* Target not detected, *cps/ml* copies/milliliter, *COR* Crude odd ratio, *AOR* Adjusted odd ratio, *CI* confidence interval

** statistically significant, ETB− Ethiopian Birr

Multivariate analysis was done to determine the further association of the potential confounding factors such as sex, age, educational status, marital status, income, presence of latrine, viral load, and World Health Organization (WHO) stage with intestinal parasitosis. As a result, viral load level, WHO stage 4 of HIV/AIDS (the severely symptomatic stage) (17), and availability of latrine showed significant association. People living with HIV who had viral load count 20–1000 cps/ml were more likely to develop a parasitic infection than those having a viral load count results of target not detected (TND) (AOR = 2.37, 95% CI 1.92, 20.1) and those who did not have latrine were 1.2 times more likely acquire intestinal parasite infection than those who had latrine (AOR = 1.21, 95% CI 1.1, 3.4). Patients who had WHO stage 4 of HIV/AIDS were more likely infected with parasitic infection than those who had stage 1. (AOR = 3.83, 95% CI 1.23, 11.54) (Table 2).

## Discussion

The present study investigated the socio-demographic characteristics and prevalence of IPs among individuals living with HIV/AIDS in East and West Gojam Zones, Amhara region, Ethiopia. The findings of this study provide important insights into the factors associated with IP infections in this specific geographic area.

The socio-demographic characteristics of the study population revealed that most participants were urban residents (52%), aged between 21 and 40 (54.2%), and 38.2% couldn’t read and write, while 31.8% had completed high school. This finding is consistent with previous reports in different parts of Ethiopia. The higher rates of HIV/AIDS in urban areas due to factors such as increased mobility, higher population density, and greater access to healthcare services, and the global HIV/AIDS epidemiology reported that young adults are often at higher risk of HIV infection due to behavioral factors, including engaging in risky sexual behaviors and substance abuse. Low educational attainment is often associated with limited health literacy, which can hinder individuals' ability to understand and adopt preventive measures against parasitic infections (18–23).

The overall prevalence of IPs among people living with HIV/AIDS attending the study areas was 19.3%. This finding is consistent with some previous studies conducted in Amhara region, Ethiopia, which have reported a high burden of IP infections among HIV-positive individuals (19, 24). However, our finding was much lower than the expected prevalence obtained from the systematic review and meta-analysis research in Ethiopia (39.15%) (14). This might be due to in the study area people living with HIV/AIDS have a strong adherence to ART drugs, counseling, improved knowledge through health education, and good sanitation practices. The prevalence of specific parasites in this study revealed that *hookworms* were the most prevalent (33.3%), followed by *E. histolytica* (17%). These findings are consistent with the literature, as *hookworm* infection is known to be highly prevalent in Ethiopia, particularly in rural areas with poor sanitation and hygiene practices (25).

The association analysis between intestinal parasitosis and socio-demographic and other risk factors revealed several important findings. Female participants had a higher likelihood of being infected with intestinal parasites compared to males. This finding is consistent with previous studies in Ethiopia, which have reported a higher prevalence of intestinal parasites among females living with HIV/AIDS (26). This might be due to biological factors like immunosuppression and gastrointestinal changes, socially limited access to healthcare, stigma, and discrimination associated with HIV/AIDS, poor nutritional status, and cultural like menstrual hygiene practices and traditional practices (herbal medicine usage) differences in hygiene practices, may contribute to this gender disparity. The presence of a latrine/toilet was found to be a significant protective factor against intestinal parasitic infections. Participants who did not have access to a latrine were 1.2 times more likely to acquire such infections compared to those who had access. This finding highlights the importance of proper sanitation and hygiene practices in preventing parasitic infections, particularly in resource-limited settings like East and West Gojam Zones. Lack of access to adequate sanitation facilities increases the risk of fecal–oral transmission of parasites (27, 28).

Furthermore, the viral load level and the WHO stage of HIV/AIDS were significantly associated with intestinal parasitosis. Individuals with a viral load count between 20 and 1000 cps/ml were more likely to develop parasitic infections compared to those with undetectable viral load counts. This finding suggests that individuals with higher viral loads may have compromised immune systems, making them more susceptible to opportunistic infections, including intestinal parasites (23, 29). Additionally, patients in WHO stage 4 of HIV/AIDS had a higher likelihood of being infected with parasitic infections compared to those in stage 1. Advanced HIV/AIDS disease progression weakens the immune system, increasing vulnerability to various infections, including parasitic infections (30).

Individuals with a CD4 count below 200 cell/mm^3^, indicating advanced HIV/AIDS progression, had a significantly higher likelihood of being infected with intestinal parasites. In this study, the adjusted odds ratio was 5.7 (95% CI 2.77–11.7). Even individuals with a CD4 count between 200 and 500 cells/mm^3^ showed an increased risk of parasitic infections compared to those with higher CD4 counts, with an adjusted odds ratio of 4.6 (95% CI 1.80–11.7, p-value = 0.001). Walking barefoot was also significantly associated with a higher risk of parasitic infections, with an adjusted odds ratio of 6.6 (95% CI 2.7–16.4, p-value = 0.001). These findings emphasize the importance of monitoring CD4 counts, promoting preventive measures, and improving hygiene practices, including the use of footwear, to reduce the burden of intestinal parasitic infections among individuals living with HIV/AIDS.

The findings of this study have important implications for public health interventions in East and West Gojam Zones, Amhara region, Ethiopia. Targeted interventions should focus on improving health literacy and promoting proper sanitation and hygiene practices among individuals living with HIV/AIDS. Efforts to increase awareness about the importance of regular screening and appropriate treatment for intestinal parasitic infections are crucial. Integration of interventions targeting both HIV/AIDS and parasitic infections is recommended to improve the overall health outcomes of individuals living with HIV/AIDS. This can include providing comprehensive healthcare services that address both HIV/AIDS management and the prevention and treatment of parasitic infections. The strength of this study was used different parasitological diagnostic modalities to detect IPs in people living with HIV/AIDS, however, there was a delayance in sample transportation to the reference laboratory which performed formol-ether concentration technique and modified acid-fast staining, this issue could affect the prevalence of IPs among the participants.

## Conclusion

This study highlights the high prevalence of intestinal parasitic infections among individuals living with HIV/AIDS in East and West Gojam Zones, Amhara region, Ethiopia. The findings underscore the importance of addressing socio-demographic factors, such as gender and educational status, as well as improving sanitation and hygiene practices among this vulnerable population. Integrated interventions that target both HIV/AIDS and parasitic infections are essential to improve the overall health and well-being of individuals living with HIV/AIDS in this region.

## Data Availability

The datasets used and/or analysed during the current study are in the manuscript and available from the corresponding author on reasonable request.

## Abbreviations

AFB: Acid fast bacilli
AIDS: Acquired immuno deficiency syndrome
ART: Anti retro viral treatment
CD4: Cluster for differentiation
DMSCH: Debre markos compressive specialized hospital
FGH: Finote selam general hospital
LDH: Lumamie distirict hospital
YDH: Yejuba district hospital
CNS: Central nervous system
HIV: Human immunodeficiency virus
PLHIV: People living with human immunodeficiency virus
HTLV: Human thymus lymphocyte virus
IP: Intestinal parasite
MOH: Ministry of Health
SPSS: Stastical package of social science
RPM: Revolution per minute
UNADIS: United Nation on ADIS Program
HAART: Highly active antiretroviral therapy
PLWHA: People living with HIV/ADIS
